# Comprehensive Analysis of the Complete Mitochondrial Genome of *Paeonia ludlowii* Reveals a Dual-Circular Structure and Extensive Inter-Organellar Gene Transfer

**DOI:** 10.3390/biology14070854

**Published:** 2025-07-14

**Authors:** Zhefei Zeng, Zhengyan Zhang, Ngawang Norbu, Ngawang Bonjor, Xin Tan, Shutong Zhang, Norzin Tso, Junwei Wang, La Qiong

**Affiliations:** 1 Key Laboratory of Biodiversity and Environment on the Qinghai-Tibetan Plateau, Ministry of Education, School of Ecology and Environment, Xizang University, Lhasa 850000, China; zengzhefei@126.com (Z.Z.);; 2 Yani Observation and Research Station for Wetland Ecosystem of the Tibet (Xizang) Autonomous Region, Xizang University, Nyingchi 860000, China; 3 Ministry of Education Key Laboratory for Biodiversity Science and Ecological Engineering, Institute of Biodiversity Science, School of Life Sciences, Fudan University, Shanghai 200438, China

**Keywords:** *Paeonia ludlowii*, mitochondrial genome, repetitive sequences, phylogeny, RNA editing

## Abstract

*Paeonia ludlowii*, a critically endangered species endemic to Tibet, China, possesses significant ornamental, culinary, and medicinal value. However, its mitochondrial genome remains understudied, limiting insights into its evolutionary mechanisms and constraining conservation genetics applications and molecular breeding programs. We present the first complete assembly and comprehensive analysis of the *P. ludlowii* mitochondrial genome. Most remarkably, we discovered that the *P. ludlowii* mitogenome exhibits an atypical dual-circular structure, representing the first documented occurrence of this architectural feature within the genus *Paeonia*. The assembled genome spans 314,371 bp and encodes 42 tRNA genes, 3 rRNA genes, and 31 protein-coding genes, with a pronounced adenine–thymine bias. This multipartite genome structure is characterized by abundant repetitive elements (112 functionally annotated SSRs, 33 tandem repeats, and 945 dispersed repeats), which potentially drive genome rearrangements and facilitate adaptive evolution. Analyses of codon usage bias and nucleotide diversity revealed highly conserved gene expression regulation with limited variability. Phylogenetic reconstruction confirms that *P. ludlowii*, *P. suffruticosa*, and *P. lactiflora* form a monophyletic clade, reflecting close evolutionary relationships, while extensive syntenic collinearity with other *Paeonia* species underscores mitochondrial genome conservation at the genus level. Extensive inter-organellar gene transfer events, particularly from chloroplast to mitochondrion, suggest that such DNA exchanges enhance genetic diversity and promote environmental adaptation. The discovery of the dual-circular architecture provides novel insights into plant mitochondrial genome evolution and structural plasticity. This study elucidates the unique structural characteristics of the *P. ludlowii* mitochondrial genome and establishes a crucial genetic foundation for developing targeted conservation strategies and facilitating molecular-assisted breeding programs for this endangered species.

## Simple Summary

We present the first complete genetic map of the mitochondrion from *Paeonia ludlowii*, a critically endangered peony species found only in Tibet, China. This precious species has important ornamental, food, and medicinal uses, but its genetic makeup was previously unknown, limiting conservation efforts and breeding programs. Our study revealed that this peony has a unique dual-circular mitochondrial genome structure—essentially, two connected genetic circles instead of the typical single circle found in related peony species. This genome is exceptionally large, containing numerous repetitive genetic sequences that likely contribute to the plant’s ability to adapt to harsh mountain environments. Most remarkably, we discovered that half of the mitochondrial genome consists of genetic material transferred from the chloroplast, representing one of the most extensive examples of genetic exchange between cellular compartments ever documented in flowering plants. These transferred genes may enhance the plant’s survival capabilities in extreme conditions. Our findings provide crucial genetic information for developing targeted conservation strategies to protect this precious endangered species and establish a foundation for breeding programs to create hardier ornamental peonies. This research also advances our understanding of how plant cells evolve and exchange genetic material to survive environmental challenges.

## 1. Introduction

Mitochondria, as essential organelles in eukaryotic cells, play a central role not only in energy metabolism but also in regulating plant growth, development, and adaptation to environmental stresses [1,2]. Compared with animal mitochondria, plant mitochondrial genomes exhibit unique characteristics, including larger genome sizes, highly complex gene arrangements, and frequent structural rearrangements and inversions [3,4]. These distinctive features stem from the evolutionary trajectory of the plant mitochondrial genomes following an endosymbiotic event, resulting in significant structure and size differences compared to animal mitochondrial genomes [5]. Plant mitochondrial genome sizes vary substantially across Viridiplantae, ranging from 42 kb in the early diverging green alga *Mesostigma viride* [6] to 11.7 Mb in the gymnosperm *Larix sibirica* [7]. This extensive size variation is primarily attributed to the frequent recombination of repetitive sequences and the incorporation of foreign DNA through intracellular or intercellular horizontal gene transfer events [4,8]. These repetitive sequences not only influence the genome size, they also play crucial role in maintaining genome stability and promoting diversity, particularly in the formation of multichromosomal architectures and sub-genomic molecules [8,9]. Consequently, frequent structural rearrangements can induce dynamic changes in non-coding regions and may trigger transitions between different genome organizations, such as multichromosomal and linear configurations, through homologous recombination mechanisms [10,11].

Plant mitochondrial genomes, despite their characteristically large sizes and structural complexity, exhibit remarkably low mutation rates compared to their nuclear and chloroplast counterparts [9,12]. This exceptionally low mutation rate renders mitochondrial DNA particularly valuable for phylogenetic and evolutionary analyses, as the highly conserved mitochondrial protein-coding genes provide reliable molecular markers for elucidating plant evolutionary relationships [3,13]. Furthermore, while mitochondrial genomes display significant interspecific variation in gene number and chromosomal arrangement, the core functional genes have remained remarkably stable across extended evolutionary timescales, reflecting a strong selective pressure to maintain essential mitochondrial functions [9,14].

Assembling plant mitochondrial genomes has historically been challenging due to their complex architectures and chloroplast contamination [9,15]. Early short-read platforms have often failed to span repetitive regions, resulting in fragmented assemblies [16]. Long-read sequencing technologies (Oxford Nanopore, PacBio) have revolutionized this field by enabling the resolution of complex genome structures and revealing dynamic rearrangements in multichromosomal mitochondrial genomes [16,17]. Despite the comprehensive sequencing of numerous chloroplast genomes, mitochondrial genome studies—particularly among diverse angiosperms—remain limited [9,15]. Expanding research in this domain is essential in understanding plant genome evolution and environmental adaptation mechanisms.

*Paeonia ludlowii* (Stern & G. Taylor) J.J. Li & D.Z. Chen is a critically endangered endemic peony species restricted to the Nyingchi region of Tibet on the Qinghai–Tibetan Plateau [18]. With only six known natural populations, this species faces a severe extinction risk due to its extremely limited distribution and increasing anthropogenic pressures, resulting in its classification as a National Key Protected Wild Plant and designation as a Plant Species with Extremely Small Populations (PSESP) in China [19]. The species typically inhabits sparse montane forests and shrublands on granite substrates at high elevations, representing a unique evolutionary lineage adapted to harsh plateau environments [20]. *P. ludlowii* possesses exceptional ecological and horticultural significance: its distinctive large yellow flowers and tree-like growth form make it an invaluable genetic resource for ornamental peony breeding programs [21,22]. Additionally, this species exhibits remarkable edible and medicinal properties—its protein- and vitamin-rich petals have been traditionally used in pastry preparation since the Song Dynasty [23,24], while its seeds contain abundant phenolic antioxidants that may confer protective effects against cardiovascular disease, stroke, and certain malignancies [25].

The taxonomic status of *P. ludlowii* has been subject to ongoing debate. Originally described as a variant of *P. lutea*, Li et al. argued for its recognition as a distinct species based on morphological, reproductive, and karyotypic evidence [26]. This view was subsequently validated by Hong et al., who formally established *P. ludlowii* as an independent species within *Paeonia* [20]. Recent advances in genomic technologies have accelerated the molecular research on peonies. Zhao et al. used RAD-sequencing to analyze genome-wide SNPs and revealed low but variable genetic diversity, subtle population structures, and historical declines in *P. ludlowii*, providing important insights for its conservation [22]. Moreover, the complete 10.3 Gb nuclear genome of *P. ludlowii* has been sequenced at chromosome-level resolution, revealing substantial genomic differentiation from other peony species [21]. Additionally, comparative analyses of the chloroplast genomes of three *Paeonia* species, including *P. ludlowii*, have identified molecular markers and clarified phylogenetic relationships within the genus [27].

However, mitochondrial genome resources for *Paeonia* remain extremely limited. Currently, only two peony mitogenomes are available: Tang et al. assembled the complete mitogenome of *P. lactiflora* as a circular molecule using Nanopore sequencing and conducted preliminary phylogenetic placement within the APG IV system [28], while a mitochondrial sequence for *P. suffruticosa* (203,077 bp, NC_084129) has been deposited in NCBI. The mitogenome of *P. ludlowii* remains uncharacterized, representing a significant gap in understanding the organellar genome evolution within Paeoniaceae. Mitochondrial genome analysis provides crucial insights into cytoplasmic inheritance patterns and enables the identification of chimeric open reading frames associated with cytoplasmic male sterility (CMS), which is fundamental in developing male-sterile lines in hybrid breeding programs [15]. Understanding the molecular basis of CMS could substantially enhance peony breeding efficiency through improved crossbreeding systems.

In this study, we assembled and comprehensively characterized the complete mitochondrial genome of *P. ludlowii* for the first time. We systematically analyzed the gene content, repetitive sequence architecture, codon usage patterns, and nucleotide diversity. We also conducted comparative analyses with other Paeoniaceae mitogenomes to elucidate phylogenetic relationships and investigate inter-organellar DNA transfer events. This research advances our understanding of organellar genome evolution in *P. ludlowii* and establishes a foundation for molecular breeding applications and conservation strategies for this critically endangered species.

## 2. Materials and Methods

### 2.1. Sample and Sequencing Data Source

The mitochondrial and chloroplast genomes of *P. ludlowii* were assembled using publicly available HiFi sequencing data from Xiao et al. [21]. The original sequencing was performed using Pacific Biosciences (PacBio) Circular Consensus Sequencing (CCS) technology, generating high-quality long reads with lengths exceeding 10 kb.

Plant samples were collected from the National Tibetan Plateau Crop Germplasm Garden (Lhasa, Tibet, China) at 3600 m elevation. Total genomic DNA was extracted from young leaf tissue, and high-molecular-weight libraries were constructed using SMRTbell Express Template Prep Kit 2.0 and sequenced on a PacBio Sequel II platform (Pacific Biosciences, Menlo Park, CA, USA). We obtained the complete HiFi dataset and performed de novo assembly specifically for organellar genomes. Detailed sequencing statistics are provided in Table S1.

### 2.2. Mitochondrial Genome Assembly and Annotation

We performed the de novo assembly of high-quality PacBio HiFi reads (>10 kb in length) using the PacBio Multiplexed Assembly Tool (PMAT v1.5.3) [29] with default parameters. The resulting assembly graph was visualized using Bandage v0.8.1 [30]. To ensure assembly purity, we checked that all assembled contigs were of mitochondrial origin by performing BLASTN v2.10.1 [31] searches against the *P. ludlowii* chloroplast genome and a comprehensive plant nuclear genome database, systematically removing any contigs exhibiting high sequence similarity (>90% identity) to chloroplast or nuclear DNA. The dual-circular structure of the mitochondrial genome was confirmed through two-step validation. First, potential circular configurations were resolved based on Bandage assembly graph visualization, identifying repeat-mediated recombination sites. Second, the complete 15 Gb HiFi dataset was mapped against resolved genome structures to analyze coverage depth patterns. Continuous coverage without zero-depth regions validated the structural accuracy and confirmed the correct resolution of repeat-mediated nodes while excluding erroneously incorporated chloroplast or nuclear sequences.

For genome annotation, we employed a multi-reference approach using mitochondrial genomes from closely related *Paeonia* species (including *P. suffruticosa*) and *Arabidopsis thaliana* as templates. Protein-coding genes (PCGs) were annotated using GeSeq [32] with BLAST searches employing an E-value threshold of 1 × 10^−5^ against curated organellar gene databases, supplemented by the Plant Mitochondrial Genome Annotation tool (PMGA; http://47.96.249.172:16084/home). Transfer RNA genes were predicted using tRNAscan-SE v2.0.7 [33], while ribosomal RNA genes were identified through BLASTN v2.10.1 [31] searches against known plant rRNA sequences. All preliminary annotations were subjected to manual curation and validation using Apollo v1.11.8 [34] to ensure annotation accuracy and consistency. A comprehensive circular genome map was generated using OGDRAW v1.3.1 (https://chlorobox.mpimp-golm.mpg.de/OGDraw.html, accessed on 15 August 2024).

For comparative analyses, the *P. ludlowii* chloroplast genome was assembled using the Organelle Assembly Toolkit (OATK v1.0) and annotated with CPGAVAS2 [35]. The final curated chloroplast and mitochondrial genome sequences have been deposited in GenBank (http://www.ncbi.nlm.nih.gov) under accession numbers PQ145547 and PQ180366-PQ180367, respectively.

### 2.3. Repetitive Sequence Analysis

Three types of repetitive sequences were identified in the *P. ludlowii* mitochondrial genome: simple sequence repeats (SSRs), tandem repeats, and dispersed repeats. The MISA online tool (https://webblast.ipk-gatersleben.de/misa/, accessed on 10 March 2025) [36] was utilized to detect SSRs with minimum repeat numbers set as follows: mononucleotides ≥ 10, dinucleotides ≥ 5, trinucleotides ≥ 4, tetranucleotides ≥ 3, and pentanucleotides ≥ 3. Tandem Repeats Finder v4.09 (http://tandem.bu.edu/trf/trf.submit.options.html, accessed on 22 March 2025) [37] was employed to identify tandem repeats exceeding 6 bp with a match score above 95%. Dispersed repeats were detected using BLASTN v2.10.1 [31], with search parameters set to a word size of 7 and an E-value of 1 × 10^−5^, and further filtered to eliminate tandem repeats. Circos v0.69-5 (http://circos.ca/software/download/, accessed on 2 April 2025) was used to visualize the distribution of these repetitive sequences within the mitochondrial genome.

### 2.4. Codon Usage Preference Analysis

We analyzed codon usage preferences of protein-coding genes (PCGs) from *P. ludlowii*, *P. suffruticosa*, and *P. lactiflora*. First, we extracted PCGs using Phylosuite v1.23 [38], followed by the calculation of relative synonymous codon usage (RSCU) values using MEGA v7.0.26 [39]. Finally, these RSCU values were visualized using the Bioinformatics Cloud Platform (Nanjing Jisi Huiyuan Biotechnology Co., Ltd., Nanjing, China, accessed on 5 April 2025).

### 2.5. RNA Editing Site Prediction

To identify RNA editing sites in the *P. ludlowii* mitochondrial genome, we employed the PmtREP tool from the Bioinformatics Cloud Platform (Nanjing Jisi Huiyuan Biotechnology Co., Ltd., Nanjing, China, accessed on 11 April 2025). This analysis was conducted with reference to plant mitochondrial genome-encoded proteins.

### 2.6. Homologous Fragment Analysis of Organelle Genomes

To investigate evolutionary relationships and inter-organellar gene transfer events between the mitochondrial and chloroplast genomes of *P. ludlowii*, we conducted comprehensive homologous fragment analysis. Homologous sequences were identified using BLASTN v2.10.1 [31] with stringent parameters designed to capture biologically meaningful similarities while minimizing false positives: minimum sequence identity ≥ 70% (reflecting substantial evolutionary conservation beyond random similarity), E-value ≤ 1 × 10^−5^ (ensuring statistical significance), and minimum alignment length ≥ 30 bp (corresponding to approximately 10 codons, sufficient to represent functionally relevant DNA segments and exclude spurious short matches that may arise from compositional bias or random sequence similarity).

The 70% identity threshold was selected based on previous studies on inter-organellar DNA transfer in angiosperms, where transferred sequences typically retain 60–80% similarity to their source sequences due to evolutionary divergence following transfer events. The 30 bp minimum length criterion ensures the detection of fragments large enough to potentially encode functional peptides or regulatory elements, while filtering out random matches commonly occurring in AT-rich organellar genomes. All identified homologous regions were subsequently validated through manual inspection to confirm their biological relevance. The spatial distribution and directionality of homologous sequences were visualized using Circos v0.69-5 to illustrate patterns of inter-organellar similarity and potential transfer events.

### 2.7. Ka/Ks Ratio Evaluation

To assess selective pressures on mitochondrial protein-coding genes, we conducted synonymous (Ks) and non-synonymous (Ka) substitution rate analysis across six representative Saxifragales species: *P. ludlowii* (this study), *P. suffruticosa* (NC_084129), *P. lactiflora* (NC_070189), *Ribes nigrum* (NC_082306), *Rhodiola rosea* (PP024540), and *Sedum plumbizincicola* (NC_069572).

Orthologous protein-coding sequences were identified through BLASTN v2.10.1 [31] searches and aligned using MAFFT v7.310 [40]. Alignment quality was manually inspected to exclude poorly aligned regions that could introduce artifacts. Ka/Ks ratios were calculated using the MLWL model in Ka/Ks Calculator v2.0 [41].

To validate genes showing Ka/Ks > 1, we performed sliding-window analysis (window size = 30 codons, step size = 3 codons) using DnaSP v6.12.03 [42] to identify regions under potential positive selection. The results were interpreted conservatively, with Ka/Ks > 1 considered indicative of relaxed purifying selection or potential positive selection only when supported by sliding window analysis. The results were visualized as boxplots using the R package ggplot2 in R v4.3.0.

### 2.8. Calculation of Nucleotide Diversity

Homologous gene sequences from six species were aligned using the auto mode of MAFFT v7.310 [40]. Subsequently, nucleotide diversity (Pi) values were calculated for each gene using DnaSP 6.12.03 [42].

### 2.9. Comparative Analysis of Mitochondrial Genome Structure

For comparative analysis, we utilized the six previously mentioned representative mitochondrial genomes and generated dot plots using the maxmatch parameter in MUMmer version 4.0.0beta2 [43]. This facilitated the comparison of sequence differences between the *P. ludlowii* mitochondrial genome and the selected species. Homologous sequences were identified using BLASTN v 2.10.1 [31], with parameters set to a word size of 7, an E-value threshold of 1 × 10^−5^, and a minimum length threshold of 300 bp.

### 2.10. Phylogenetic Analysis

To elucidate the phylogenetic relationships of *P. ludlowii*, we retrieved 12 closely related plant mitochondrial genomes from the NCBI database: *P. ludlowii* (this study), *P. suffruticosa* (NC_084129), *P. lactiflora* (NC_070189), *Vitis vinifera* (NC_012119), *R. alpinum* (PP716826), *R. nigrum* (NC_082306), *R. rosea* (PP024540), *R. bupleurifolia* (NC_082108), R. tangutica (NC_072122), *R. crenulata* (NC_070303), *R. sacra* (OP312070, OP312071), *R. wallichiana* (OP312068, OP312069), and *S. plumbizincicola* (NC_069572). We selected *V. vinifera* (order Vitales) as the outgroup based on its phylogenetic position outside the Saxifragales clade, providing appropriate evolutionary distance for robust tree rooting.

All shared conserved protein-coding genes (PCGs) were extracted from the mitochondrial genomes using PhyloSuite v1.2.3 [38] and aligned using MAFFT v7.310 [40] with default parameters. To ensure optimal phylogenetic inference, we employed jModelTest v2.1.10 [44] to determine the best-fitting nucleotide substitution model, which identified GTR + I + G as the most appropriate model based on the corrected Akaike Information Criterion (AICc). Maximum likelihood phylogenetic reconstruction was performed using IQ-TREE v1.6.12 [45] with 1000 bootstrap replicates to assess branch support. The resulting phylogenetic tree was visualized and formatted using FigTree v1.4.4 (http://tree.bio.ed.ac.uk/software/figtree/, accessed on 19 April 2025).

## 3. Results

### 3.1. Mitochondrial Genome Structure and Content

**Figure 1:**
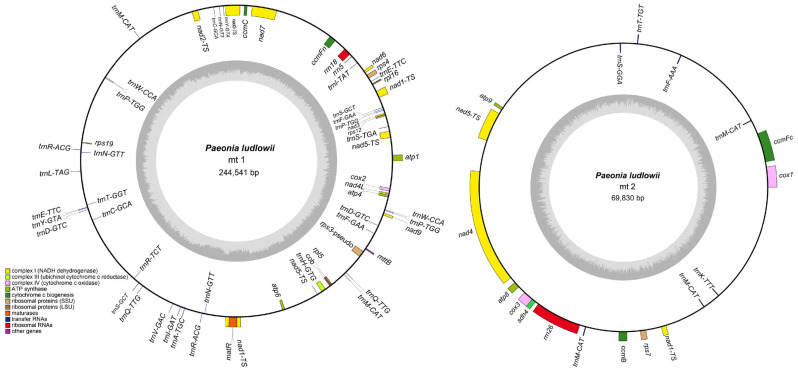
Mitochondrial genome map of *P. ludlowii*. Genes transcribed in the forward direction are displayed on the outer rim of the circle, while those transcribed in the reverse direction are presented on the inner rim. The innermost gray circle illustrates the GC content distribution across the genome.

Based on assembly graph analysis (Figure S1), we successfully assembled the *P. ludlowii* mitochondrial genome with a total length of 314,371 bp organized into two distinct circular DNA molecules (Figure 1), representing the first dual-circular mitochondrial genome reported in Paeoniaceae. The assembly showed high coverage depth (>500×) across all mitochondrial contigs, indicating high assembly accuracy. To validate the structural continuity, we mapped a subset of 15 Gb HiFi reads against the assembled genome, revealing uniform coverage (~35×) without gaps (Figure S2), confirming the integrity of both circular chromosomes. The larger circular chromosome (mt1) spans 244,541 bp with a GC content of 41.72%, while the smaller circular chromosome (mt2) comprises 69,830 bp with a GC content of 44.33%. The overall GC content is 42.30%, reflecting the AT-rich characteristic typical of *P. ludlowii* mitochondrial genomes (Table 1).

Comprehensive gene annotation revealed 78 genes in the *P. ludlowii* mitochondrial genome, comprising 31 protein-coding genes (PCGs), 42 transfer RNA genes, 3 ribosomal RNA genes, and 2 pseudogenes (Table S2). Among the protein-coding genes, 25 represent core mitochondrial genes essential in oxidative phosphorylation and electron transport: 5 ATP synthase genes, 9 NADH dehydrogenase genes, 4 cytochrome c biogenesis genes, and 3 cytochrome c oxidase genes, plus single genes for transport membrane protein (*mttB*), maturase (*matR*), ubiquinol cytochrome c reductase (*cob*), and succinate dehydrogenase (*sdh4*). Additionally, six non-core genes encode ribosomal proteins (two large-subunit proteins and four small-subunit proteins). Notably, three genes—*nad1*, *nad5*, and *trnM-CAT*—were annotated in both mitochondrial circular molecules (mt1 and mt2), suggesting potential gene redundancy or structural overlap between the two molecules.

Structural analysis revealed that three tRNA genes (*trnA-TGC*, *trnI-GAT*, and *trnI-TAT*) contain a single intron. Notably, 12 genes exhibit copy number variation, all of which are tRNA genes. The *trnM-CAT* gene shows the highest copy number with five copies, while *trnN-GTT* and *trnP-TGG* each have three copies. The remaining nine multi-copy tRNA genes are present with two copies each (Table S2).

### 3.2. Analysis of Repetitive Sequences

**Figure 2:**
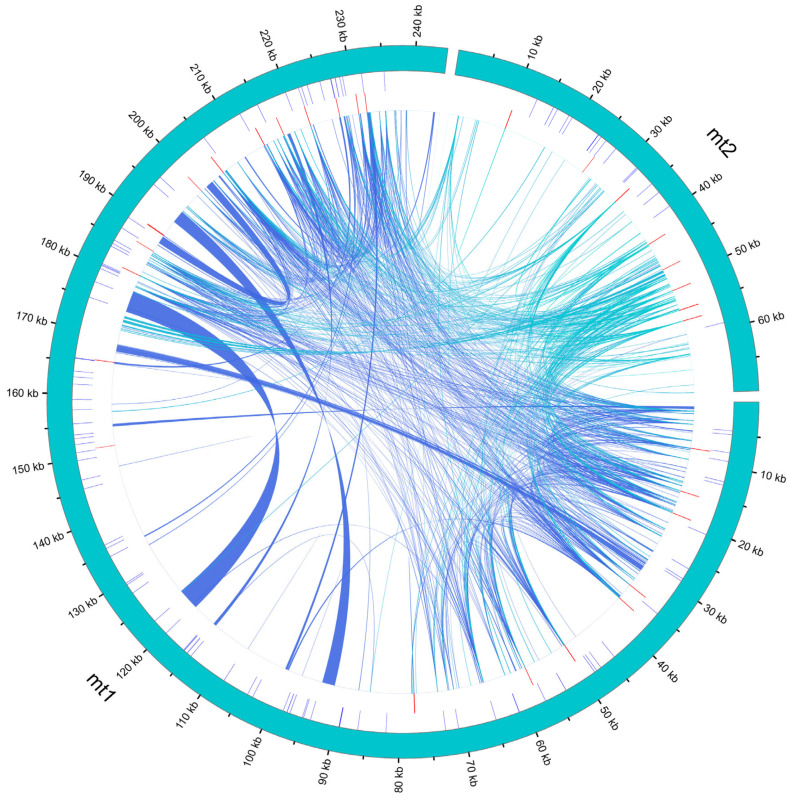
Distribution of repetitive sequences in the *P. ludlowii* mitochondrial genome. The outermost circle represents the size range of the mitochondrial genome. SSRs and tandem repeats are depicted in blue and red, respectively. The innermost circle presents the locations of dispersed repeats.

The *P. ludlowii* mitochondrial genome harbors an exceptionally rich repertoire of repetitive DNA elements, comprising 112 SSRs, 33 tandem repeats, and 945 dispersed repeats distributed throughout both circular chromosomes (Figure 2). This abundance of repetitive elements is characteristic of complex plant mitochondrial genomes and may contribute to genomic instability and structural rearrangements. To further explore the mitochondrial genome context, we analyzed the positional distribution of all repeat types relative to gene annotation. The vast majority of repeats were located in intergenic regions, with only a small proportion overlapping coding or intronic regions.

SSRs: We identified 112 SSRs with motif lengths ranging from 1 to 6 nucleotides. Mononucleotide repeats predominated (64 occurrences, 57.1%), followed by dinucleotide repeats (31 occurrences, 27.7%), while higher-order repeats (tri-, tetra-, penta-, and hexanucleotide) were less frequent (Table S3). The distribution included 82 SSRs in mt1 and 30 in mt2, reflecting the proportional size difference between the two circular chromosomes.

Tandem Repeats: Tandem repeats, also termed satellite DNA, consist of contiguous repetitions of short motifs (1–200 bp) ubiquitous in eukaryotic and some prokaryotic genomes [46,47]. Our analysis revealed 24 tandem repeats in mt1 and 9 in mt2, with repeat unit lengths ranging from 8 to 48 bp and sequence identity exceeding 70% (Table S4). The considerable sequence diversity among these tandem repeats suggests that they may serve important functional roles, potentially mediating genomic structural variation, facilitating recombination events, or influencing gene expression regulation.

Dispersed Repeats: The most abundant repetitive elements were dispersed repeats, totaling 945 sequences distributed across both chromosomes (Table S5). These repeats exhibited diverse lengths and orientations, with palindromic repeats being slightly more abundant than forward repeats across most length categories (Figure S3). Both types were especially concentrated in the 30–49 bp range. The high density and variability of these dispersed repeats may contribute to the structural complexity of the *P. ludlowii* mitochondrial genome and could facilitate the generation of alternative genomic conformations via homologous recombination.

### 3.3. Codon Usage Analysis

We analyzed codon usage preferences in the mitochondrial genomes of *P. ludlowii* and its close relatives *P. lactiflora* and *P. suffruticosa*. Among the three species, *P. ludlowii* exhibited the highest number of codons (9014), followed by *P. suffruticosa* (8913), and *P. lactiflora* (8822) (Table S6). Among all amino acids, leucine (Leu), serine (Ser), isoleucine (Ile), and glycine (Gly) were the most frequently used in all three species, while cysteine (Cys) and tryptophan (Trp) were the least frequently used, a pattern consistent with other plant mitochondrial genomes [3,48], indicating the relative conservation of the PCGs in the *P. ludlowii* mitochondrial genome (Figure S4). As shown in Table S2, most PCGs utilized ATG as the start codon; however, the *nad1* and *nad4L* genes employed ACG as the start codon, which may be related to RNA editing. For stop codons, TAA was the most commonly used, accounting for 45.16% of all stop codons, followed by TGA at 32.26%.

**Figure 3:**
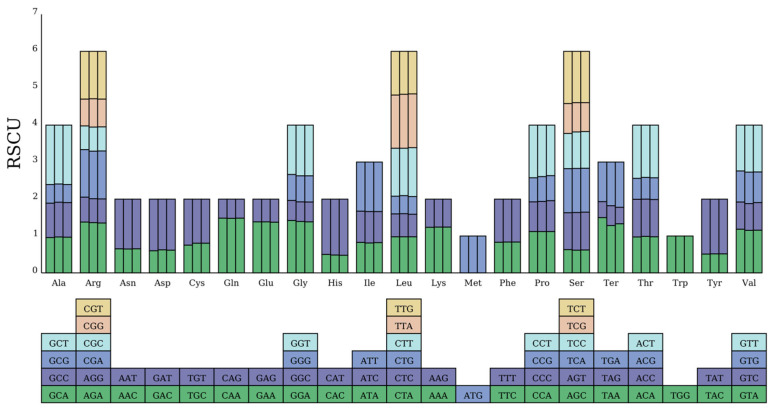
RSCU values in the mitochondrial genome of *P. ludlowii*. The *X*-axis represents various amino acids, while the *Y*-axis shows RSCU values. RSCU is a measure that quantifies the observed frequency of each codon relative to its expected frequency, assuming uniform usage among synonymous codons. For each amino acid, the three bars from left to right correspond to *P. ludlowii*, *P. lactiflora*, and *P. suffruticosa*, respectively.

To further explore codon usage preferences, we calculated the RSCU values (Table S6). An RSCU value of 1 indicates no significant codon usage preference, while values below 1 indicate that the codon is used less frequently than its synonymous counterparts, and values above 1 indicate a higher usage frequency. As shown in Figure 3, the mitochondrial PCGs of the three species exhibited preferences for specific codons. The number of codons with an RSCU greater than 1 was 5179 in *P. ludlowii*, 5188 in *P. lactiflora*, and 5243 in *P. suffruticosa*, indicating that these codons were used more frequently than their synonymous counterparts. Additionally, the majority of codons with RSCU values greater than 1 ended with an A or U base, accounting for 94.07%, 94.26%, and 94.32% of the total in the three species, respectively. This phenomenon suggests a strong A/U bias in codons with higher usage frequencies in the mitochondrial genomes of *Paeonia* species.

### 3.4. RNA Editing Prediction

**Figure 4:**
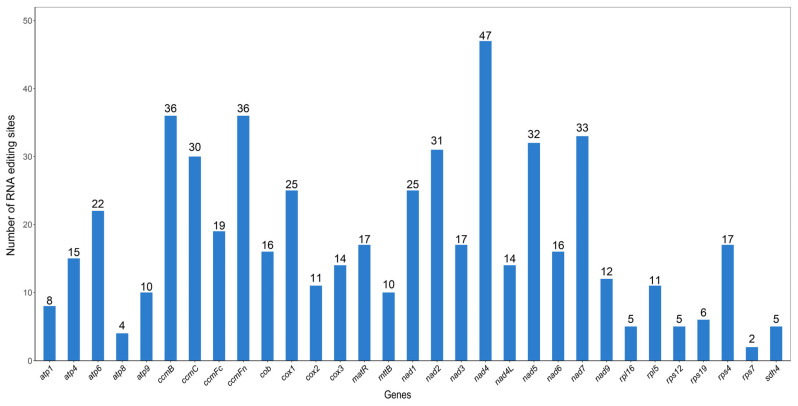
Distribution of RNA editing sites in protein-coding genes of the *P. ludlowii* mitochondrial genome.

In eukaryotes, the modification of bases in transcribed RNA coding regions through addition, deletion, or substitution is referred to as RNA editing [49,50]. This study predicted 551 RNA editing sites across the 31 PCGs of the *P. ludlowii* mitochondrial genome (Figure 4). The distribution of these sites varied considerably among genes, ranging from a minimum of 2 sites in *rps7* to a maximum of 47 sites in *nad4*. Following RNA editing, 44.83% of the amino acids maintained their hydrophobicity, while 7.99% transitioned from hydrophobic to hydrophilic and 46.46% shifted from hydrophilic to hydrophobic (Table 2).

Furthermore, 30 codon transition types involving 10 amino acid conversions were identified. Among all the codon transitions, TCA ⇒ TTA was the most common, with 78 sites. The results also demonstrated that the most frequent amino acid generated after RNA editing was Leu, with 45.19% (249 sites) of the edited amino acids being converted to Leu. All RNA editing sites in the *P. ludlowii* mitochondrial genome involved C-to-T editing. Among them, 29.95% (165 sites) of the editing events occurred at the first position of the codon and 66.06% (364 sites) at the second position (Table S7), while no editing occurred at the third position of the codon. Two unique editing events were identified, where both the first and second positions of the codon were edited, resulting in the conversion of proline (CCC, CCT) to phenylalanine (TTC, TTT). Additionally, 0.73% of the amino acids were edited into stop codons (TAG, TGA).

### 3.5. Homologous Fragment Analysis

A comparative analysis between the *P. ludlowii* mitochondrial and chloroplast genomes revealed extensive chloroplast-to-mitochondrion DNA transfer events (Table S8). We identified 33 chloroplast-derived fragments integrated into the mitochondrial genome, collectively spanning 157,428 bp and representing 50.08% of the total mitochondrial genome length—an exceptionally high proportion that far exceeds the typical 1–5% observed in most angiosperms and even surpasses the ~12% reported in *P. lactiflora* [28].

The chloroplast-derived segments exhibit remarkable size heterogeneity, ranging from short fragments of 42 bp to an extraordinarily large contiguous insertion of 35,961 bp. This largest transferred fragment displays > 99% sequence identity to the corresponding chloroplast region and encompasses multiple intact chloroplast genes, including several photosynthesis-related genes and ribosomal protein genes. The high sequence conservation and gene content integrity provide compelling evidence for authentic inter-organellar DNA transfer rather than assembly artifacts or cross-contamination during sequencing.

**Figure 5:**
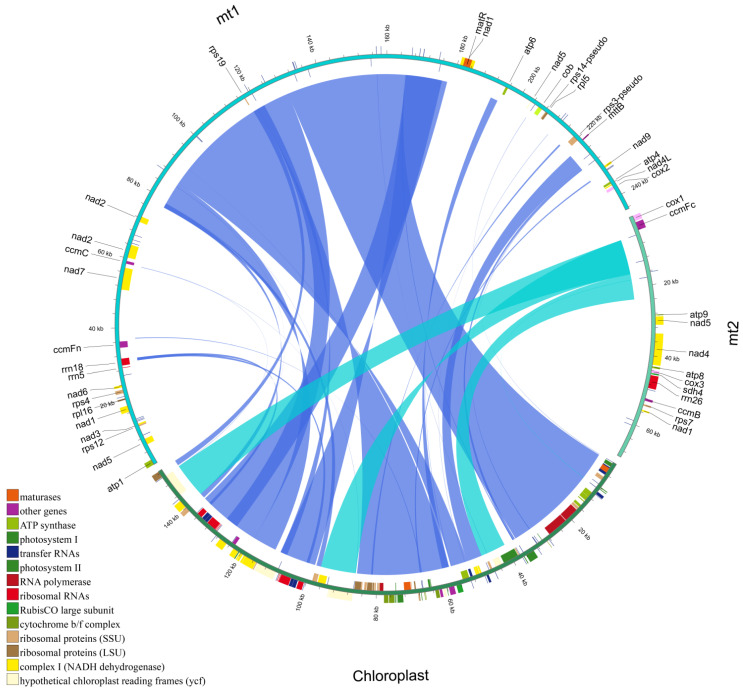
Homologous fragments between chloroplast and mitochondrial genome sequences in *P. ludlowii*. The chloroplast and mitochondrial sequences are depicted, with homologous genes from corresponding complexes highlighted in matching colors. The lines in the center illustrate homologous sequences.

Synteny analysis revealed that the vast majority of the chloroplast genome has been incorporated into the mitochondrial genome through these transfer events. Only five chloroplast genes showed no detectable sequence similarity in the mitochondrial assembly: the protein-coding genes *ycf1*, *accD*, *rps12*, and *rpl23*, and the tRNA gene *trnV-UAC* (Figure 5). This near-complete representation of chloroplast genetic material within the mitochondrial genome suggests multiple independent transfer events throughout evolutionary history.

The transferred chloroplast sequences are distributed across both mitochondrial circular chromosomes, with larger fragments predominantly located in mt1 (the major circle) and smaller fragments scattered throughout both circles. Notably, several transferred regions retain their original gene order and spacing, indicating that substantial chloroplast genomic segments were transferred as intact units rather than through piecemeal insertion events.

### 3.6. Ka/Ks Ratio Analysis

**Figure 6:**
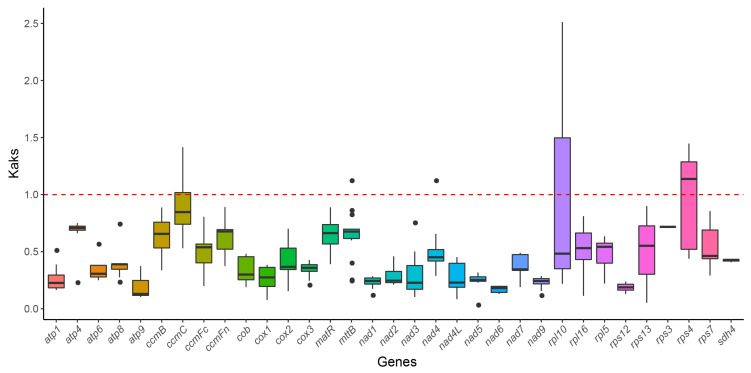
Boxplots showing pairwise Ka/Ks ratios among shared mitochondrial genome genes across 6 Saxifragales plants. Colors are used only to distinguish genes and do not carry biological significance.

To evaluate the evolutionary selective pressures on the mitochondrial PCGs of *P. ludlowii* and closely related species, we calculated the ratio of non-synonymous (Ka) to synonymous (Ks) substitutions (Ka/Ks). The Ka/Ks ratio is a metric widely used to discern the type of selective pressure acting on protein-coding genes throughout evolution. The red dashed line denotes the baseline of Ka/Ks = 1, signifying neutral selection. A Ka/Ks ratio exceeding 1 (Ka/Ks > 1) suggests positive selection, indicating the potential adaptive evolution of the gene, whereas a Ka/Ks ratio below 1 (Ka/Ks < 1) implies purifying selection, in which deleterious mutations are eliminated [51]. The results demonstrate that the Ka/Ks ratio for most genes was below 1, signifying that these genes have undergone strong purifying selection during evolution, with mutations generally not altering the amino acid sequence and thus being selectively removed (Figure 6). Notably, genes such as *atp1*, *nad6*, *atp9*, *atp6*, *cox1*, and *rps12* exhibited remarkably low Ka/Ks ratios, indicating that these genes are under stringent functional constraints. However, certain genes had Ka/Ks ratios approaching or marginally surpassing 1, suggesting potential neutral or mild positive selection. For instance, the *rpl10* and *rps4* genes had Ka/Ks ratios close to or slightly exceeding 1, which may indicate positive selection pressure on these genes in certain species, possibly associated with adaptive evolution. Variations in these genes may confer selective advantages for survival in specific ecological environments and thus be retained during evolution.

### 3.7. Comparative Nucleotide Diversity Analysis

**Figure 7:**
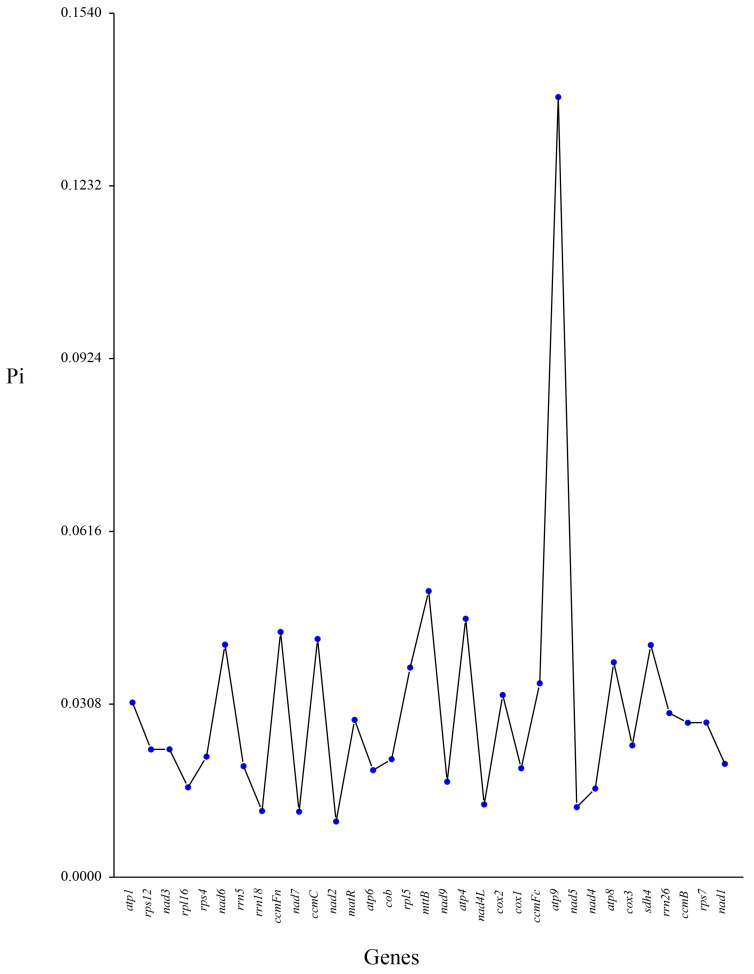
Pi across mitochondrial protein-coding genes based on genome comparisons among six Saxifragales species. The six species analyzed were *P. ludlowii, P. suffruticosa*, *P. lactiflora*, *R. nigrum*, *R. rosea*, and *S. plumbizincicola*.

Pi, a key indicator of genetic variation within a species, is utilized as a molecular marker in population genetics studies [52,53]. We conducted Pi analysis on 30 PCGs and 3 rRNA genes from *P. ludlowii* and five closely related species. The results demonstrated that most genes exhibited low Pi values, indicating high conservation among species, with few mutations and stable gene functions (Figure 7). However, the *atp9* gene displayed a significantly higher Pi value (0.13904), contrasting with its low Ka/Ks ratio, suggesting that the *atp9* gene exhibits a high level of sequence variability across species, but these variations are predominantly neutral and do not significantly alter protein function. This phenomenon may indicate that the *atp9* gene has not been subjected to strong purifying selection during evolution, allowing for the accumulation of neutral mutations and resulting in increased Pi. Additionally, genes such as *rrn18*, *nad7*, *nad2*, *nad4L*, and *nad5* exhibited low Pi values (<0.02), indicating high conservation in Saxifragales species, with few mutations. These genes likely play essential roles in maintaining basic metabolic functions, thus experiencing strong purifying selection pressure during evolution.

### 3.8. Collinearity Analysis

**Figure 8:**
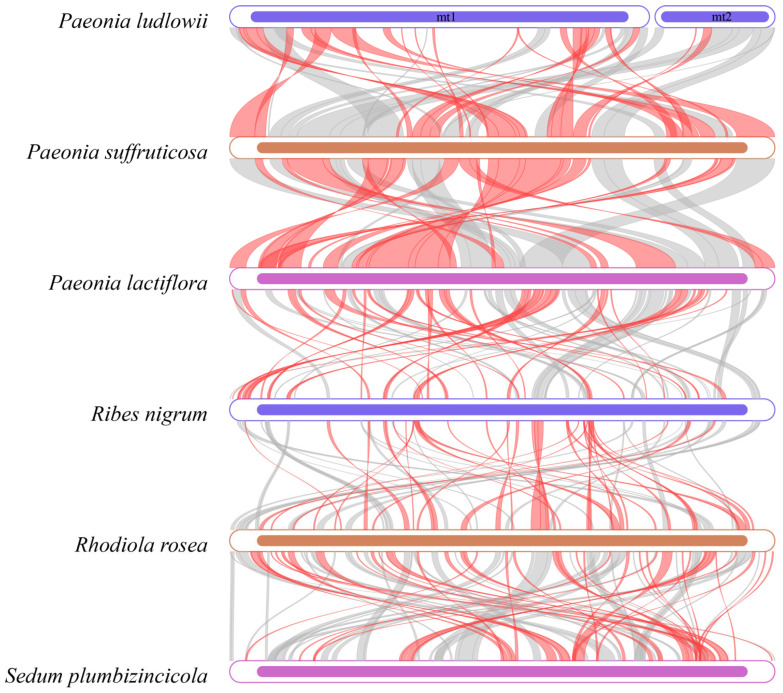
A multiple synteny plot comparing the mitochondrial genome of *P. ludlowii* with those of closely related species. Each box in a row represents a genome, and the connecting lines indicate regions with sequence similarity. Red arcs highlight inverted regions, while gray arcs denote regions with higher sequence similarity.

Dot plot analysis revealed distinct collinearity patterns at different taxonomic levels. Among the three *Paeonia* species (*P. ludlowii*, *P. suffruticosa*, and *P. lactiflora*), we observed high collinearity with extensive syntenic blocks, indicating substantial genome structural conservation within the genus (Figure S5). In contrast, the comparison of *P. ludlowii* with more distantly related Saxifragales species—including *R. nigrum* (Grossulariaceae), *R. rosea* and *S. plumbizincicola* (Crassulaceae)—revealed numerous regions with sequence similarity but extensive structural rearrangements and inconsistent syntenic organization (Figure 8). While shared genes with high sequence similarity are present across species, their relative positions and orientations differ substantially. These results demonstrate that the mitochondrial genome structure is highly conserved within *Paeonia* but shows significant reorganization between different families within Saxifragales.

### 3.9. Phylogenetic Relationships

**Figure 9:**
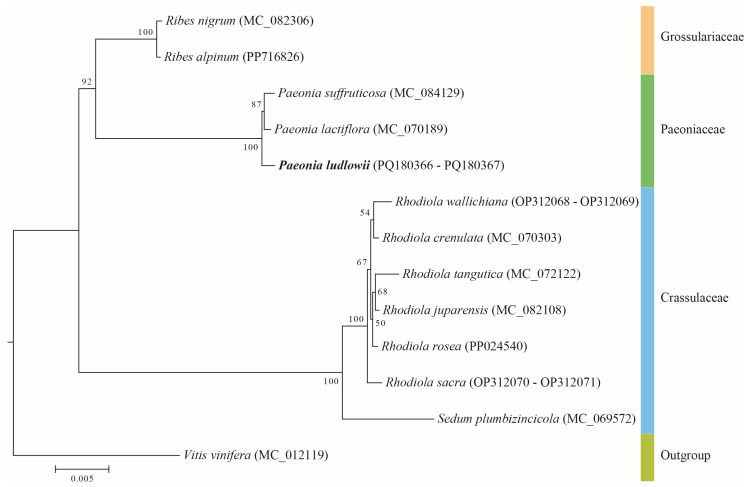
A phylogenetic tree constructed using the mitochondrial protein-coding genes of 13 species. The tree displays bootstrap support values for each node, expressed as percentages calculated from 1000 replicates. The various families are differentiated by distinct colors, with *P. ludlowii* prominently highlighted in bold.

We conducted a phylogenetic analysis of 13 species based on the DNA sequences of 29 conserved mitochondrial PCGs (Figure 9). The findings reveal that *P. ludlowii*, *P. suffruticosa*, and *P. lactiflora* constitute a monophyletic group, with *P. ludlowii* and *P. suffruticosa* exhibiting a sister group relationship with *P. lactiflora*. The phylogenetic tree also suggests a close evolutionary relationship between Paeoniaceae and Grossulariaceae, followed by Crassulaceae, which aligns with recent classification results from the APG [54].

## 4. Discussion

### 4.1. Mitogenome Architecture and Gene Content

The complete mitochondrial genome of *P. ludlowii* presents a remarkable dual-circular architecture totaling 314,371 bp, representing the first multichromosomal mitogenome reported within Paeoniaceae. This structure distinguishes *P. ludlowii* from its congeners *P. lactiflora* (~182 kb) and *P. suffruticosa* (~203 kb), which possess conventional single-circular genomes [28]. While documented in angiosperms such as *Salvia officinalis* and *Rhododendron wallichiana* [55,56], these non-canonical conformations remain relatively uncommon, likely resulting from specific evolutionary pressures or genomic instability events facilitating structural reorganization.

The genome exhibits characteristic AT bias (GC content: 42.30%) typical of plant mitochondria and encodes 78 genes, including 31 protein-coding genes with 25 core genes essential for oxidative phosphorylation. Notably, 12 tRNA genes show copy number variation, with *trnM-CAT* displaying the highest copy number (five copies). This multi-copy phenomenon is prevalent in plant mitochondrial genomes and may enhance gene expression efficiency or maintain genome stability through functional redundancy during evolutionary processes [3,52].

### 4.2. Repetitive Elements and Genome Complexity

The *P. ludlowii* mitochondrial genome exhibits an exceptionally rich repertoire of repetitive DNA elements, comprising 112 SSRs, 33 tandem repeats, and 945 dispersed repeats distributed across both circular chromosomes. This abundance significantly exceeds that observed in the closely related species *P. lactiflora* and likely contributes to the structural complexity and large size of this genome [28].

The SSR composition shows mononucleotide repeats as the predominant category (64 occurrences, 57.1%), followed by dinucleotide repeats (31 occurrences, 27.7%), primarily composed of A/T bases consistent with the genome’s AT bias. The 33 tandem repeats, with unit lengths ranging from 8 to 48 bp and sequence identities exceeding 70%, demonstrate considerable structural diversity that may facilitate genomic rearrangements and recombination events.

Most notably, the 945 dispersed repeats represent the most abundant repetitive element category, with palindromic repeats slightly predominating over forward repeats, particularly in the 30–49 bp size range. This high density of dispersed repeats likely serves as a primary driver of genomic instability and may facilitate the generation of alternative genomic conformations through homologous recombination mechanisms [46,47].

Positional analysis revealed that the vast majority of repetitive elements are preferentially located within intergenic regions rather than coding sequences. This distribution pattern reflects evolutionary constraints that minimize disruption to essential gene function, as structural variations within intergenic regions are generally less likely to compromise cellular viability compared to alterations in protein-coding sequences. Consequently, intergenic repeats serve as primary hotspots for homologous recombination events, enabling genome expansion and structural plasticity while preserving gene integrity. This organizational pattern is consistent with observations across angiosperm mitochondrial genomes, where the accumulation of intergenic repetitive elements drives genomic diversification without compromising essential cellular functions [8,9]. The exceptional abundance of repetitive elements in *P. ludlowii* therefore supports the hypothesis that repetitive element proliferation constitutes a fundamental mechanism underlying mitochondrial genome expansion and architectural evolution.

### 4.3. Codon Usage Patterns and Translation Optimization

Codon usage analysis revealed that *P. ludlowii* exhibits patterns consistent with other plant mitochondrial genomes. Among the three *Paeonia* species examined, *P. ludlowii* contains the highest codon count (9014), followed by *P. suffruticosa* (8913) and *P. lactiflora* (8822). Leucine, serine, isoleucine, and glycine were the most frequently utilized amino acids across all three species, while cysteine and tryptophan showed the lowest usage frequencies. This amino acid composition pattern is consistent with other plant mitochondrial genomes [47], reflecting evolutionary constraints on protein structure within the organellar environment. The conservation of codon usage patterns among *Paeonia* species indicates relative evolutionary stability of protein-coding genes, supporting the observed structural conservation within this genus.

RSCU analysis demonstrated a strong preference for A/U-ending codons, with 94.07% of frequently used codons (RSCU > 1) terminating in these bases. This bias reflects evolutionary optimization for translation efficiency and mRNA stability, as A/U-rich codons often correspond to more abundant tRNAs and facilitate faster translation rates [57,58]. Notably, *nad1* and *nad4L* genes employ ACG start codons, likely associated with RNA editing events that restore canonical ATG codons post-transcriptionally, ensuring proper translation initiation.

### 4.4. RNA Editing Site Prediction and Functional Implications

Computational prediction identified 551 C-to-U RNA editing sites distributed across 31 mitochondrial protein-coding genes in *P. ludlowii*, revealing an extensive post-transcriptional modification landscape characteristic of plant mitochondria. The editing sites varied considerably among genes, ranging from 2 in *rps7* to 47 in *nad4*, consistent with previous observations in rice and wheat where extensive editing ensures proper protein function [59,60]. All editing events represented C-to-U conversions, with 66.06% occurring at the second codon position, a pattern that predominantly restores conserved amino acids crucial in mitochondrial protein functionality in land plants [60]. Notably, 46.46% of predicted editing events convert hydrophilic residues to hydrophobic ones, potentially enhancing protein stability and membrane integration, with leucine being the most frequent editing product (45.19% of edited sites). Two unique double-editing events were predicted to convert proline codons (CCC, CCT) to phenylalanine (TTC, TTT), representing rare instances of extensive codon modification within plant mitochondria.

However, the computational nature of these predictions necessitates experimental validation to confirm their biological relevance. As emphasized in recent studies, only transcript-level evidence obtained through methods such as transcriptome-wide RNA sequencing or RT-PCR can definitively establish the in vivo occurrence and physiological impact of RNA editing events in plant mitochondria [59]. While these computational predictions provide valuable insights into the potential RNA editing landscape of *P. ludlowii*, experimental validation through transcriptomic analysis remains essential to confirm actual editing events and their functional significance in mitochondrial protein regulation and plant adaptation.

### 4.5. Inter-Organellar DNA Transfer and Genomic Evolution

Our analysis revealed exceptional chloroplast-to-mitochondrion DNA transfer in *P. ludlowii*, with 33 transferred fragments collectively spanning 157,428 bp and representing 50.08% of the total mitogenome length. This proportion far exceeds the typical 1–5% observed in most angiosperms [61,62] and even surpasses the ~12% reported in *P. lactiflora* [28], suggesting particularly active inter-organellar genetic exchange in this lineage.

The transferred sequences exhibit remarkable size heterogeneity, ranging from 42 bp to an extraordinarily large 35,961 bp fragment that retains >99% sequence identity to the corresponding chloroplast region. The presence of intact chloroplast genes within these transferred segments, combined with high sequence conservation, provides compelling evidence for authentic inter-organellar DNA transfer rather than assembly artifacts. Such extensive transfer events may contribute to evolutionary adaptation by introducing chloroplast-derived genetic material that can enhance metabolic flexibility or stress tolerance, though the functional validation of these transferred sequences requires transcriptomic analysis to determine their expression status and potential contributions to mitochondrial function.

### 4.6. Selective Pressures and Evolutionary Constraints

Ka/Ks analysis across six Saxifragales species revealed that most mitochondrial protein-coding genes are under strong purifying selection (Ka/Ks < 1), reflecting functional constraints on essential respiratory components. Critical genes including *atp1*, *nad6*, *atp9*, *atp6*, *cox1*, and *rps12* exhibited remarkably low Ka/Ks ratios, indicating evolutionary conservation essential in maintaining protein function across species. This pattern is consistent with observations in *Spartina alterniflora* and *Colobanthus* species, where core mitochondrial genes also experience pervasive purifying selection [63,64]. However, *rpl10* and *rps4* displayed Ka/Ks ratios approaching or exceeding unity, suggesting potential lineage-specific adaptive pressures that merit further phylogenetic investigation.

Pi analysis corroborated these evolutionary patterns, with most genes exhibiting low π values indicative of strong conservation. Core respiratory genes including *rrn18*, *nad7*, *nad2*, *nad4L*, and *nad5* displayed π values below 0.02, underscoring their essential roles in mitochondrial function. Notably, *atp9* exhibited elevated nucleotide diversity (π = 0.13904) despite its low Ka/Ks ratio, suggesting a tolerance for synonymous mutations that preserve protein function while allowing sequence divergence. This pattern highlights the selective pressures operating on mitochondrial genomes, where structural constraints maintain functional sequences while permitting neutral evolutionary changes.

### 4.7. Phylogenetic Analysis and Genome Structural Evolution

Phylogenetic analysis based on 29 conserved mitochondrial protein-coding genes strongly supports the monophyly of *Paeonia*, with *P. ludlowii* forming a well-supported clade with *P. suffruticosa* and *P. lactiflora*. The sister group relationship between *P. ludlowii* and *P. suffruticosa*, distinct from *P. lactiflora*, is consistent with previous morphological and molecular studies [22]. The phylogenetic topology also confirms the close evolutionary relationship between Paeoniaceae and Grossulariaceae within Saxifragales, followed by Crassulaceae, aligning with current APG IV classification [54].

Collinearity analysis reveals taxonomically correlated patterns in genome structural evolution that closely parallel phylogenetic relationships. Within the genus *Paeonia*, dot plot comparisons demonstrate exceptional structural conservation, with *P. ludlowii*, *P. suffruticosa*, and *P. lactiflora* exhibiting extensive syntenic blocks and high collinearity. This remarkable genome stability within *Paeonia* contrasts sharply with the structural dynamics observed at higher taxonomic levels. Comparative analysis with more distantly related Saxifragales species—including *R. nigrum* (Grossulariaceae), *R. rosea*, and *S. plumbizincicola* (Crassulaceae)—reveals numerous homologous regions with high sequence similarity, yet these are accompanied by extensive structural rearrangements and inconsistent syntenic organization.

The integration of phylogenetic and structural analyses provides mechanistic insights into mitochondrial genome evolution and adaptive diversification. Conserved protein-coding genes serve as molecular chronometers that record evolutionary history through sequence divergence patterns, while simultaneously acting as anchors that reveal genome rearrangement events through their altered positional relationships across lineages. The differential preservation of gene order within *Paeonia* versus extensive reorganization between families demonstrates how genome rearrangements accumulate proportionally with evolutionary time and taxonomic distance. These rearrangements, including inversions, translocations, and duplications, create opportunities for functional innovation by altering gene expression contexts and regulatory networks [46,47]. Furthermore, the presence of numerous repetitive elements in *P. ludlowii* provides recombination hotspots that facilitate both intragenome rearrangements and potential horizontal gene transfer events from other cellular compartments, contributing to the genome plasticity that is essential in adapting to diverse ecological niches. The correlation between structural complexity and environmental adaptation suggests that genome architecture itself represents an evolutionary toolkit, where rearrangement events enable functional optimization for lineage-specific ecological requirements while conserved gene content maintains essential metabolic functions.

### 4.8. Study Limitations and Future Research Directions

Several important limitations warrant acknowledgment. The 551 predicted RNA editing sites remain computationally inferred and require experimental validation through transcriptomic sequencing to confirm their occurrence and functional significance. The dual-circular genome structure, while strongly supported by read coverage analysis and junction mapping, would benefit from PCR amplification across predicted circle junctions to provide definitive structural confirmation. Additionally, potential positive selection signals in *rpl10* and *rps4* genes are based on limited taxonomic sampling and require validation with broader species representation to distinguish true positive selection from relaxed purifying selection.

Despite these limitations, our findings provide significant contributions to plant mitochondrial genomics and practical applications. The complete *P. ludlowii* mitogenome serves as a valuable reference for identifying cytoplasmic male sterility-associated genes, crucial in developing hybrid breeding systems in ornamental peonies. The extensive inter-organellar DNA transfer events and repetitive element diversity revealed in this study enhance our understanding of plant genome evolution and may inform conservation strategies by identifying genetic factors underlying species adaptability to environmental challenges. Future research incorporating transcriptomic analysis, expanded taxonomic sampling, and functional studies on transferred chloroplast sequences will further elucidate the evolutionary and adaptive significance of the unique genomic features discovered in *P. ludlowii*.

## 5. Conclusions

This study presents the first complete mitochondrial genome of *P. ludlowii*, revealing a unique dual-circular architecture totaling 314,371 bp—the first multichromosomal mitogenome reported in Paeoniaceae. The genome contains 78 genes (31 protein-coding, 42 tRNA, 3 rRNA, and 2 pseudogenes) with characteristic AT bias and exceptional repetitive sequence abundance (1090 elements), contributing to its structural complexity and large size relative to other *Paeonia* species.

Comparative genomic analysis revealed extensive inter-organellar DNA transfer, with chloroplast-derived sequences comprising 50.08% of the mitogenome—significantly higher than other published angiosperm species. This substantial transfer may represent an important evolutionary mechanism, though functional validation through transcriptomic analysis remains necessary. Collinearity analysis demonstrated high structural conservation within *Paeonia* while revealing extensive rearrangements compared to other Saxifragales families.

Evolutionary analyses identified most protein-coding genes under purifying selection, with *rpl10* and *rps4* showing potential positive selection signals that require broader taxonomic validation. Computational prediction identified 551 RNA editing sites across protein-coding genes, though experimental verification is essential to confirm these findings. Phylogenetic analysis confirmed *P. ludlowii*’s placement within a monophyletic *Paeonia* clade and supported current taxonomic relationships within Saxifragales.

This research establishes a valuable genomic resource for *Paeonia* breeding programs, particularly in identifying cytoplasmic male sterility-related genes crucial to hybrid development. The findings also provide insights into mitochondrial genome dynamics that may inform conservation strategies by revealing genetic factors underlying species adaptability. While acknowledging limitations, including the need for transcriptomic validation and broader phylogenetic sampling, this study advances our understanding of plant mitochondrial genome evolution and establishes a foundation for both fundamental research and practical applications in *Paeonia* genetics and breeding.

## Figures and Tables

**Table 1:** Accession number for the mitogenome of *P. ludlowii*.

Genome	Genome Type	Genome Length	Genome GC (%)	CDS	tRNA	rRNA	Pseudo Gene
mt1	circular	244,541	41.72	22	35	2	2
mt2	circular	69,830	44.33	9	7	1	0
Total	-	314,371	42.3	31	42	3	2

**Table 2:** The prediction of RNA editing sites in the mitogenome of *P. ludlowii*.

Type	RNA Editing	Number	Percentage
hydrophilic–hydrophilic	CAC (H) ⇒ TAC (Y)	7	
	CAT (H) ⇒ TAT (Y)	18	
	CGC (R) ⇒ TGC (C)	13	
	CGT (R) ⇒ TGT (C)	28	
	Total	66	11.98%
hydrophilic–hydrophobic	ACA (T) ⇒ ATA (I)	5	
	ACC (T) ⇒ ATC (I)	1	
	ACG (T) ⇒ ATG (M)	6	
	ACT (T) ⇒ ATT (I)	6	
	CGG (R) ⇒ TGG (W)	29	
	TCA (S) ⇒ TTA (L)	78	
	TCC (S) ⇒ TTC (F)	35	
	TCG (S) ⇒ TTG (L)	42	
	TCT (S) ⇒ TTT (F)	54	
	Total	256	46.46%
hydrophilic–stop	CAG (Q) ⇒ TAG (X)	1	
	CGA (R) ⇒ TGA (X)	3	
	Total	4	0.73%
hydrophobic–hydrophilic	CCA (P) ⇒ TCA (S)	9	
	CCC (P) ⇒ TCC (S)	12	
	CCG (P) ⇒ TCG (S)	5	
	CCT (P) ⇒ TCT (S)	18	
	Total	44	7.99%
hydrophobic–hydrophobic	CCA (P) ⇒ CTA (L)	51	
	CCC (P) ⇒ CTC (L)	12	
	CCC (P) ⇒ TTC (F)	8	
	CCG (P) ⇒ CTG (L)	39	
	CCT (P) ⇒ CTT (L)	27	
	CCT (P) ⇒ TTT (F)	14	
	CTC (L) ⇒ TTC (F)	8	
	CTT (L) ⇒ TTT (F)	14	
	GCC (A) ⇒ GTC (V)	1	
	GCG (A) ⇒ GTG (V)	5	
	GCT (A) ⇒ GTT (V)	2	
	Total	181	32.85%
	All	551	100%

## Data Availability

The chloroplast genome supporting this study has been deposited in GenBank (http://www.ncbi.nlm.nih.gov) under the accession number PQ145547, and the mitochondrial genome has been deposited under accession numbers PQ180366–PQ180367. Additionally, the HiFi sequencing data of *P. ludlowii* are available in the China National GeneBank Database under accession number CRA011243 and can be accessed at https://ngdc.cncb.ac.cn/gsa/browse/CRA011243/CRR793857 (accessed on 10 August 2024).

## Supplementary Materials

The following supporting information can be downloaded at https://www.mdpi.com/article/10.3390/biology14070854/s1. Figure S1: Preliminary assembly draft of the mitochondrial genome of *P. ludlowii.* Figure S2: Depth coverage plots of the two mitochondrial genome isoforms (mt1 and mt2) of *P. ludlowii*, based on 15 Gb of sequencing data. No uncovered regions (depth = 0) were observed in either isoform. Regions with notably high coverage correspond to chloroplast-derived insertions. Figure S3: Distribution of lengths of interspersed repeats in the *P. ludlowii* mitogenome. Figure S4: Codon usage frequency by amino acid across species. *: stop codon. Figure S5: Dot plot of *P. ludlowii* with closely related species. The horizontal coordinate in each box indicates the assembled sequence, the vertical coordinate indicates the other sequences, the red line in the box indicates the forward comparison, and the blue line indicates the reverse complementary comparison. Table S1: Basic information of HiFi sequencing data for *P. ludlowii*. Table S2: Functional classifications and physical locations of genes in the mitochondrial genome of *P. ludlowii*. Table S3: Distribution of SSRs in the mitochondrial genome of *P. ludlowii*. Table S4: Distribution of tandem repeats in the mitochondrial genome of *P. ludlowii*. Table S5: Distribution of dispersed repeats in the mitochondrial genome of *P. ludlowii*. Table S6: Codon usage of amino acid pairs in the mitochondrial genomes of three species. Table S7: Prediction of RNA editing sites in the mitochondrial genome of *P. ludlowii*. Table S8: Homologous fragments between mitochondria and chloroplasts in *P. ludlowii.*

## References

[1] Mackenzie S., McIntosh L. (1999). Higher plant mitochondria. Plant Cell.

[2] Barreto P., Koltun A., Nonato J., Yassitepe J., Maia I.D.G., Arruda P. (2022). Metabolism and signaling of plant mitochondria in adaptation to environmental stresses. Int. J. Mol. Sci..

[3] Zeng Z., Zhang Z., Tso N., Zhang S., Chen Y., Shu Q., Li J., Liang Z., Wang R., Wang J. (2024). Complete mitochondrial genome of Hippophae tibetana: Insights into adaptation to high-altitude environments. Front. Plant Sci..

[4] Wynn E.L., Christensen A.C. (2019). Repeats of unusual size in plant mitochondrial genomes: Identification, incidence and evolution. G3 Genes Genomes Genet..

[5] Lang B.F., Gray M.W., Burger G. (1999). Mitochondrial genome evolution and the origin of eukaryotes. Annu. Rev. Genet..

[6] Turmel M., Otis C., Lemieux C. (2022). The complete mitochondrial DNA sequence of Mesostigma viride identifies this green alga as the earliest green plant divergence and predicts a highly compact mitochondrial genome in the ancestor of all green plants. Mol. Biol. Evol..

[7] Putintseva Y.A., Bondar E.I., Simonov E.P., Sharov V.V., Oreshkova N.V., Kuzmin D.A., Konstantinov Y.M., Shmakov V.N., Belkov V.I., Sadovsky M.G. (2020). Siberian larch (Larix sibirica Ledeb.) mitochondrial genome assembled using both short and long nucleotide sequence reads is currently the largest known mitogenome. BMC Genom..

[8] Xiong A.S., Peng R.H., Zhuang J., Gao F., Zhu B., Fu X.Y., Xue Y., Jin X.F., Tian Y.S., Zhao W. (2008). Gene duplication and transfer events in plant mitochondria genome. Biochem. Biophys. Res. Commun..

[9] Møller I.M., Rasmusson A.G., Van Aken O. (2021). Plant mitochondria–past, present and future. Plant J..

[10] Sloan D.B. (2013). One ring to rule them all? Genome sequencing provides new insights into the ‘master circle’model of plant mitochondrial DNA structure. New Phytol..

[11] Yang Z., Ni Y., Lin Z., Yang L., Chen G., Nijiati N., Hu y., Chen X. (2022). De novo assembly of the complete mitochondrial genome of sweet potato (Ipomoea batatas [L.] Lam) revealed the existence of homologous conformations generated by the repeat-mediated recombination. BMC Plant Biol..

[12] Christensen A.C. (2013). Plant mitochondrial genome evolution can be explained by DNA repair mechanisms. Genome Biol. Evol..

[13] Liu G., Cao D., Li S., Su A., Geng J., Grover C.E., Hu S., Hua J. (2013). The complete mitochondrial genome of Gossypium hirsutum and evolutionary analysis of higher plant mitochondrial genomes. PLoS ONE.

[14] Maréchal A., Brisson N. (2010). Recombination and the maintenance of plant organelle genome stability. New Phytol..

[15] Wang J., Kan S., Liao X., Zhou J., Tembrock L.R., Daniell H., Jin S., Wu Z. (2024). Plant organellar genomes: Much done, much more to do. Trends Plant Sci..

[16] Yuan Y., Bayer P.E., Batley J., Edwards D. (2017). Improvements in genomic technologies: Application to crop genomics. Trends Biotechnol..

[17] Lang D., Zhang S., Ren P., Liang F., Sun Z., Meng G., Tan Y., Li X., Lai Q., Han L. (2020). Comparison of the two up-to-date sequencing technologies for genome assembly: HiFi reads of Pacific Biosciences Sequel II system and ultralong reads of Oxford Nanopore. Gigascience.

[18] Yu H., Deane D.C., Sui X., Fang S., Chu C., Liu Y., He F. (2019). Testing multiple hypotheses for the high endemic plant diversity of the Tibetan Plateau. Glob. Ecol. Biogeogr..

[19] Yang J., Cai L., Liu D., Chen G., Gratzfeld J., Sun W. (2020). China’s conservation program on plant species with extremely small populations (PSESP): Progress and perspectives. Biol. Conserv..

[20] Hong D.Y., Zhou S.L., He X.J., Yuan J.H., Zhang Y.L., Cheng F.Y., Zeng X.L., Wang Y., Zhang X.X. (2017). Current status of wild tree peony species with special reference to conservation. Biodivers. Sci..

[21] Xiao P.X., Li Y., Lu J., Zuo H., Pingcuo G., Ying H., Zhao F., Xu Q., Zeng X., Jiao W.B. (2023). High-quality assembly and methylome of a Tibetan wild tree peony genome (Paeonia ludlowii) reveal the evolution of giant genome architecture. Hortic. Res..

[22] Zhao Y.J., Yin G.S., Gong X. (2023). RAD-sequencing improves the genetic characterization of a threatened tree peony (Paeonia ludlowii) endemic to China: Implications for conservation. Plant Divers..

[23] Li C., Du H., Wang L., Shu Q., Zheng Y., Xu Y., Zhang J., Zhang J., Yang R., Ge Y. (2009). Flavonoid composition and antioxidant activity of tree peony (Paeonia section Moutan) yellow flowers. J. Agric. Food Chem..

[24] Zhang C.Q., Xu Y.J., Lu Y.Z., Li L.Q., Lan X.Z., Zhong Z.C. (2020). Study on the fatty acids, aromatic compounds and shelf life of Paeonia ludlowii kernel oil. J. Oleo Sci..

[25] Zhang X.X., Shi Q.Q., Ji D., Niu L.X., Zhang Y.L. (2017). Determination of the phenolic content, profile, and antioxidant activity of seeds from nine tree peony (Paeonia section Moutan DC) species native to China. Food Res. Int..

[26] Li J.J., Chen D.Z., Yu L., He L.X., Chen X.L. (1998). A study on taxonomic position of Paeonia ludlowii. Bull. Bot. Res..

[27] Cai H., Xu R., Tian P., Zhang M., Zhu L., Yin T., Zhang H., Liu X. (2023). Complete Chloroplast Genomes and the Phylogenetic Analysis of Three Native Species of Paeoniaceae from the Sino-Himalayan Flora Subkingdom. Int. J. Mol. Sci..

[28] Tang P., Ni Y., Li J., Lu Q., Liu C., Guo J. (2024). The Complete Mitochondrial Genome of Paeonia lactiflora Pall. (Saxifragales: Paeoniaceae): Evidence of Gene Transfer from Chloroplast to Mitochondrial Genome. Genes.

[29] Bi C., Shen F., Han F., Qu Y., Hou J., Xu K., Xu L.A., He W., Wu Z., Yin T. (2024). PMAT: An efficient plant mitogenome assembly toolkit using low-coverage HiFi sequencing data. Hortic. Res..

[30] Wick R.R., Schultz M.B., Zobel J., Holt K.E. (2015). Bandage: Interactive visualization of de novo genome assemblies. Bioinformatics.

[31] Chen Y., Ye W., Zhang Y., Xu Y. (2015). High speed BLASTN: An accelerated MegaBLAST search tool. Nucleic Acids Res..

[32] Tillich M., Lehwark P., Pellizzer T., Ulbricht-Jones E.S., Fischer A., Bock R., Greiner S. (2017). GeSeq–versatile and accurate annotation of organelle genomes. Nucleic Acids Res..

[33] Chan P.P., Lin B.Y., Mak A.J., Lowe T.M. (2021). tRNAscan-SE 2.0: Improved detection and functional classification of transfer RNA genes. Nucleic Acids Res..

[34] Lee E., Harris N., Gibson M., Chetty R., Lewis S. (2009). Apollo: A community resource for genome annotation editing. Bioinformatics.

[35] Shi L., Chen H., Jiang M., Wang L., Wu X., Huang L., Liu C. (2019). CPGAVAS2, an integrated plastome sequence annotator and analyzer. Nucleic Acids Res..

[36] Beier S., Thiel T., Münch T., Scholz U., Mascher M. (2017). MISA-web: A web server for microsatellite prediction. Bioinformatics.

[37] Benson G. (1999). Tandem repeats finder: A program to analyze DNA sequences. Nucleic Acids Res..

[38] Zhang D., Gao F., Jakovlić I., Zou H., Zhang J., Li W.X., Wang G.T. (2020). PhyloSuite: An integrated and scalable desktop platform for streamlined molecular sequence data management and evolutionary phylogenetics studies. Mol. Ecol. Resour..

[39] Kumar S., Stecher G., Tamura K. (2016). MEGA7: Molecular evolutionary genetics analysis version 7.0 for bigger datasets. Mol. Biol. Evol..

[40] Katoh K., Standley D.M. (2013). MAFFT multiple sequence alignment software version 7: Improvements in performance and usability. Mol. Biol. Evol..

[41] Wang D., Zhang Y., Zhang Z., Zhu J., Yu J. (2010). KaKs_Calculator 2.0: A toolkit incorporating gamma-series methods and sliding window strategies. GPB.

[42] Rozas J., Ferrer-Mata A., Sánchez-DelBarrio J.C., Guirao-Rico S., Librado P., Ramos-Onsins S.E., Sánchez-Gracia A. (2017). DnaSP 6: DNA sequence polymorphism analysis of large data sets. Mol. Biol. Evol..

[43] Marçais G., Delcher A.L., Phillippy A.M., Coston R., Salzberg S.L., MUMmer A.Z. (2018). A fast and versatile genome alignment system. PLoS Comput. Biol..

[44] Darriba D., Taboada G.L., Doallo R., Posada D. (2012). jModelTest 2: More models, new heuristics and high-performance computing. Nat. Methods.

[45] Nguyen L.T., Schmidt H.A., Von Haeseler A., Minh B.Q. (2015). IQ-TREE: A fast and effective stochastic algorithm for estimating maximum-likelihood phylogenies. Mol. Biol. Evol..

[46] Mehrotra S., Goyal V. (2014). Repetitive sequences in plant nuclear DNA: Types, distribution, evolution and function. GPB.

[47] Gualberto J.M., Mileshina D., Wallet C., Niazi A.K., Weber-Lotfi F., Dietrich A. (2014). The plant mitochondrial genome: Dynamics and maintenance. Biochimie.

[48] Li X., Han Q., Li M., Luo Q., Zhu S., Zheng Y., Tan G. (2023). Complete Mitochondrial Genome Sequence, Characteristics, and Phylogenetic Analysis of Oenanthe javanica. Agronomy.

[49] Small I.D., Schallenberg-Rüdinger M., Takenaka M., Mireau H., Ostersetzer-Biran O. (2020). Plant organellar RNA editing: What 30 years of research has revealed. Plant J..

[50] Knoop V. (2023). C-to-U and U-to-C: RNA editing in plant organelles and beyond. J. Exp. Bot..

[51] Hurst L.D. (2002). The Ka/Ks ratio: Diagnosing the form of sequence evolution. Trends Genet..

[52] Zhou P., Zhang Q., Li F., Huang J., Zhang M. (2023). Assembly and comparative analysis of the complete mitochondrial genome of Ilex metabaptista (Aquifoliaceae), a Chinese endemic species with a narrow distribution. BMC Plant Biol..

[53] Dwiningsih Y., Rahmaningsih M., Alkahtani J. (2020). Development of single nucleotide polymorphism (SNP) markers in tropical crops. Adv. Sustain. Sci. Eng. Technol..

[54] Chase M.W., Christenhusz M.J., Fay M.F., Byng J.W., Judd W.S., Soltis D.E., Mabberley D.J., Sennikov A.N., Soltis P.S., Angiosperm Phylogeny Group (2016). An update of the Angiosperm Phylogeny Group classification for the orders and families of flowering plants: APG IV. Bot. J. Linn. Soc..

[55] Yang H., Chen H., Ni Y., Li J., Cai Y., Wang J., Liu C. (2023). Mitochondrial genome sequence of Salvia officinalis (Lamiales: Lamiaceae) suggests diverse genome structures in cogeneric species and finds the stop gain of genes through RNA editing events. Int. J. Mol. Sci..

[56] Yu X., Wei P., Chen Z., Li X., Zhang W., Yang Y., Liu C., Zhao S., Li X., Liu X. (2023). Comparative analysis of the organelle genomes of three Rhodiola species provide insights into their structural dynamics and sequence divergences. BMC Plant Biol..

[57] Zhang Y., Ma Y., Yu H., Han Y., Yu T. (2024). Deciphering Codon Usage Patterns in the Mitochondrial Genome of the Oryza Species. Agronomy.

[58] Dang J., Liu Z., Luo X., Jiang Y., Zhang Z., Abdullah S., Yusop M.R. (2024). Codon Usage Characteristics and Evolutionary Analysis of Mitochondrial Genome of Winter Squash (Cucurbita maxima Duch.). J. Biobased Mater. Bioenergy.

[59] Zheng P., Wang D., Huang Y., Chen H., Du H., Tu J. (2020). Detection and analysis of C-to-U RNA editing in rice mitochondria-encoded ORFs. Plants.

[60] Gualberto J.M., Lamattina L., Bonnard G., Weil J.H., Grienenberger J.M. (1989). RNA editing in wheat mitochondria results in the conservation of protein sequences. Nature.

[61] Wang L., Liu X., Xu Y., Zhang Z., Wei Y., Hu Y., Zheng C., Qu X. (2024). Assembly and comparative analysis of the first complete mitochondrial genome of a traditional Chinese medicine Angelica biserrata (Shan et Yuan) Yuan et Shan. Int. J. Biol. Macromol..

[62] Zeng Z., Mao C., Shang Z., Norbu N., Bonjor N., Jia X., Li W., Zhang W., Wang J., Qiong L. (2025). Assembly and Comparative Analysis of the Complete Mitochondrial Genome of Hippophae salicifolia. Biology.

[63] Zhu H., Yue C., Li H. (2025). Mitochondrial Genome Characteristics and Comparative Genomic Analysis of Spartina alterniflora. Curr. Issues Mol. Biol..

[64] Androsiuk P., Paukszto Ł., Jastrzębski J.P., Milarska S.E., Okorski A., Pszczółkowska A. (2022). Molecular diversity and phylogeny reconstruction of genus Colobanthus (Caryophyllaceae) based on mitochondrial gene sequences. Genes.

