# Supplementary material for: Comprehensive Analysis of the Complete Mitochondrial Genome of Paeonia ludlowii Reveals a Dual-Circular Structure and Extensive Inter-Organellar Gene Transfer
Source: Biology (Basel). 2025 Jul 14;14(7):854. doi: 10.3390/biology14070854 (PMC12292509; doi:10.3390/biology14070854)
Supplement: Supplementary file 1 [file biology-14-00854-s001.zip › Supplementary Material Figures.pdf]

Supplementary Material

Comprehensive analysis of the complete mitochondrial genome of Paeonia ludlowii reveals a dual-circular structure and extensive inter-organellar gene transfer

Zhefei Zeng[1,2], Zhengyan Zhang[3], Ngawang Norbu[1], Ngawang Bonjor[1], Xin Tan[1], Shutong Zhang[1], Norzin Tso[1], Junwei Wang[1,2*], La Qiong[1,2*]

*: Correspondence: Junwei Wang (jwyx12240315@126.com) and La Qiong (lhagchong@163.com)

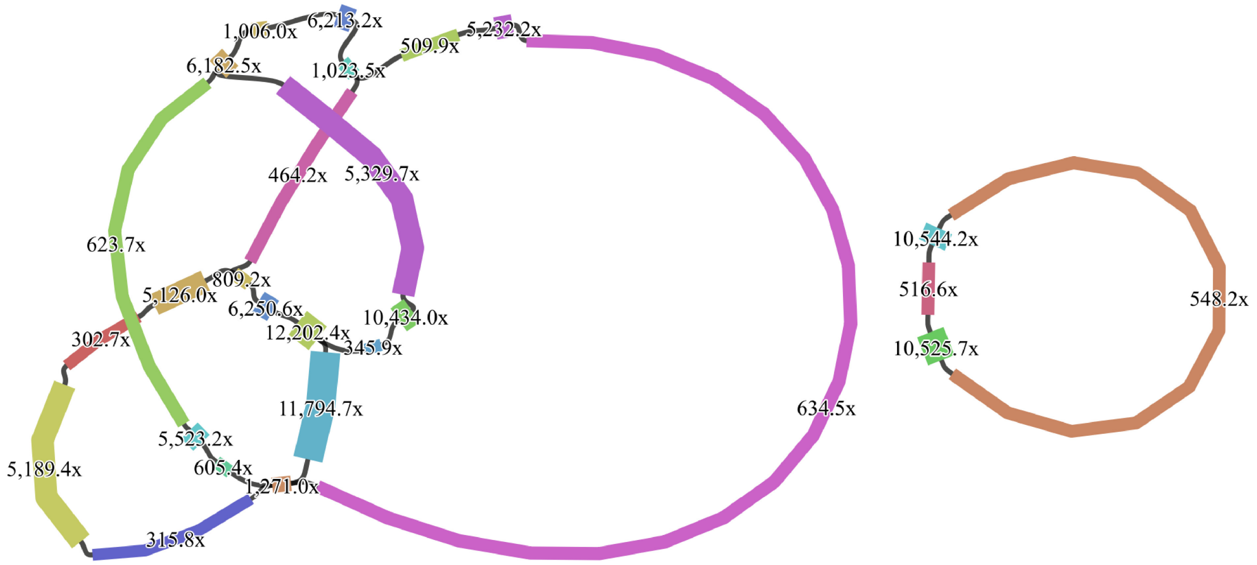

Figure S1 Preliminary assembly draft of the mitochondrial genome of *P. ludlowii*

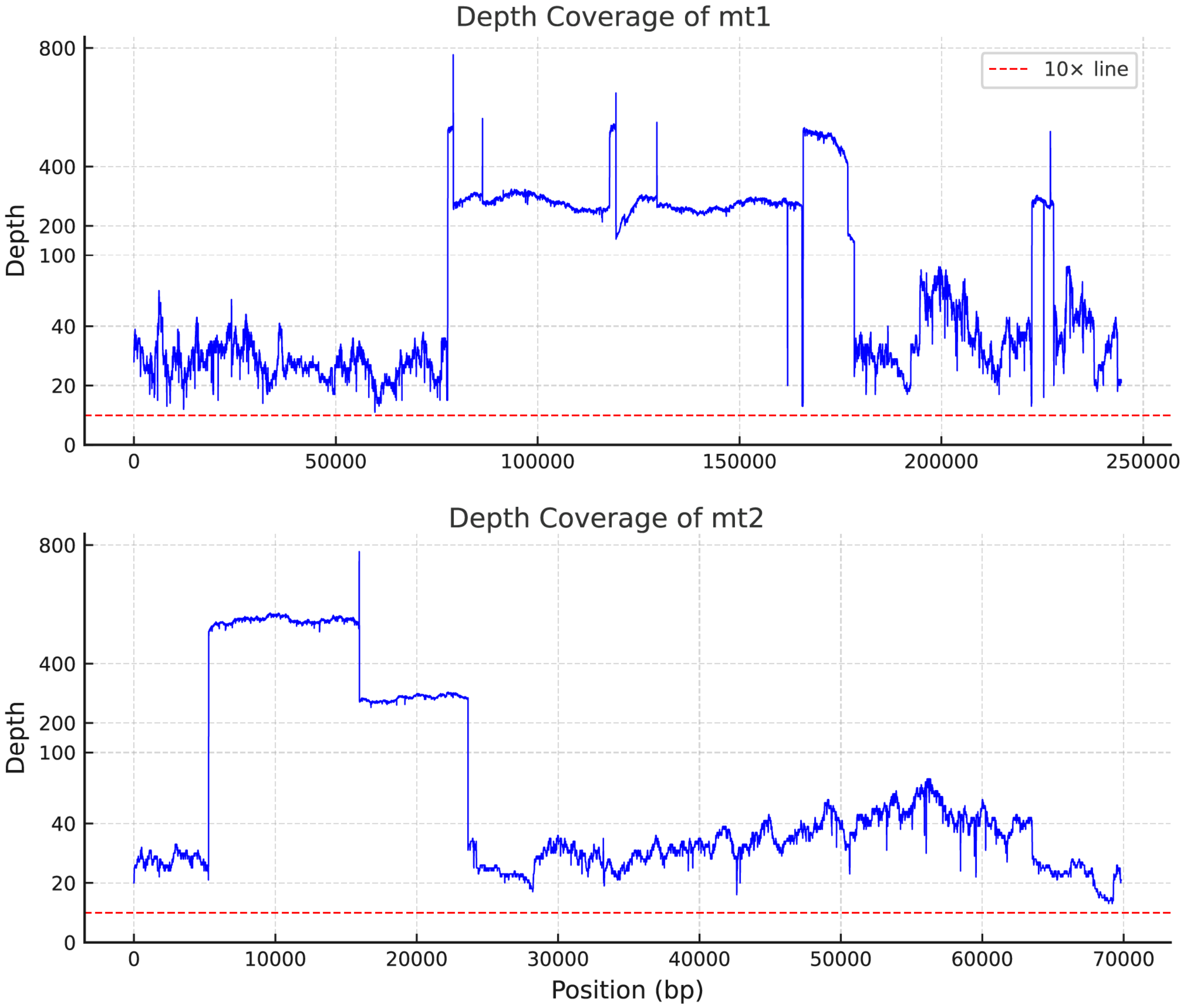

Figure S2 Depth coverage plots of the two mitochondrial genome isoforms (mt1 and mt2) of *P. ludlowii*, based on 15 Gb of sequencing data. No uncovered regions (depth = 0) were observed in either isoform. Regions with notably high coverage correspond to chloroplast-derived insertions.

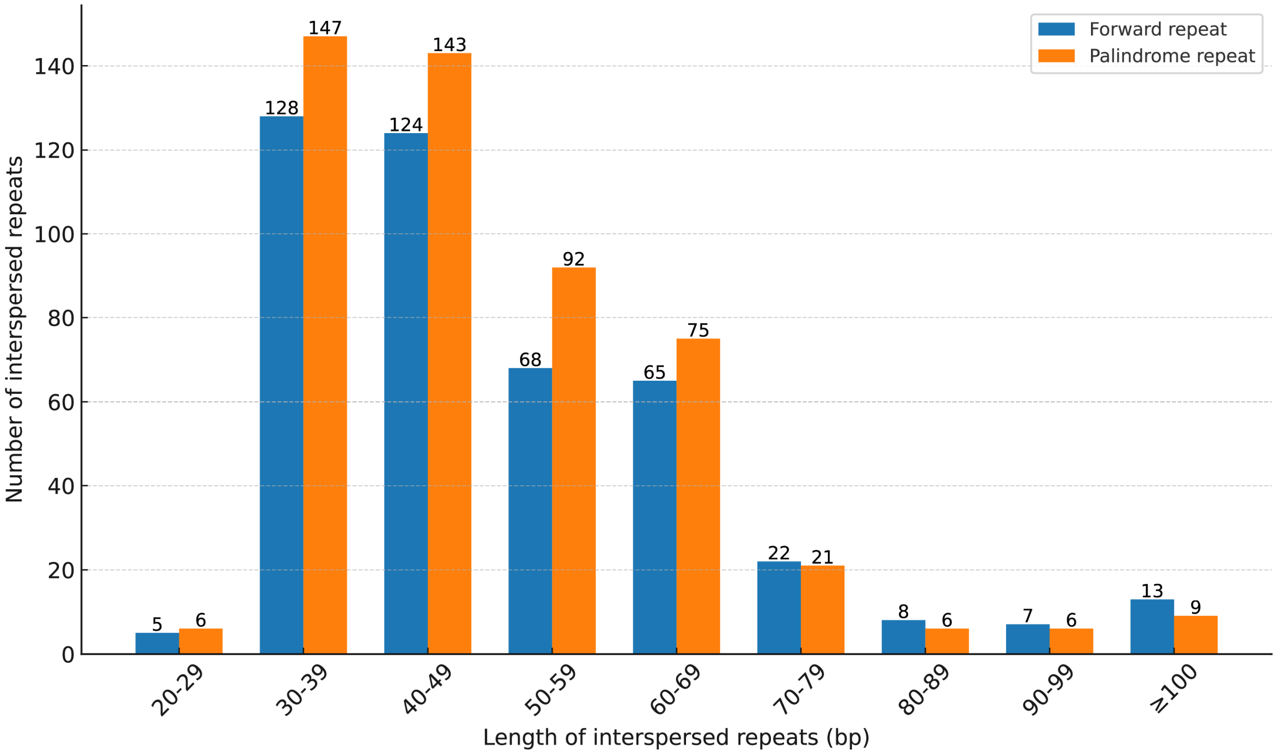

Figure S3 Distribution of lengths of interspersed repeats in the *P. ludlowii* mitogenome.

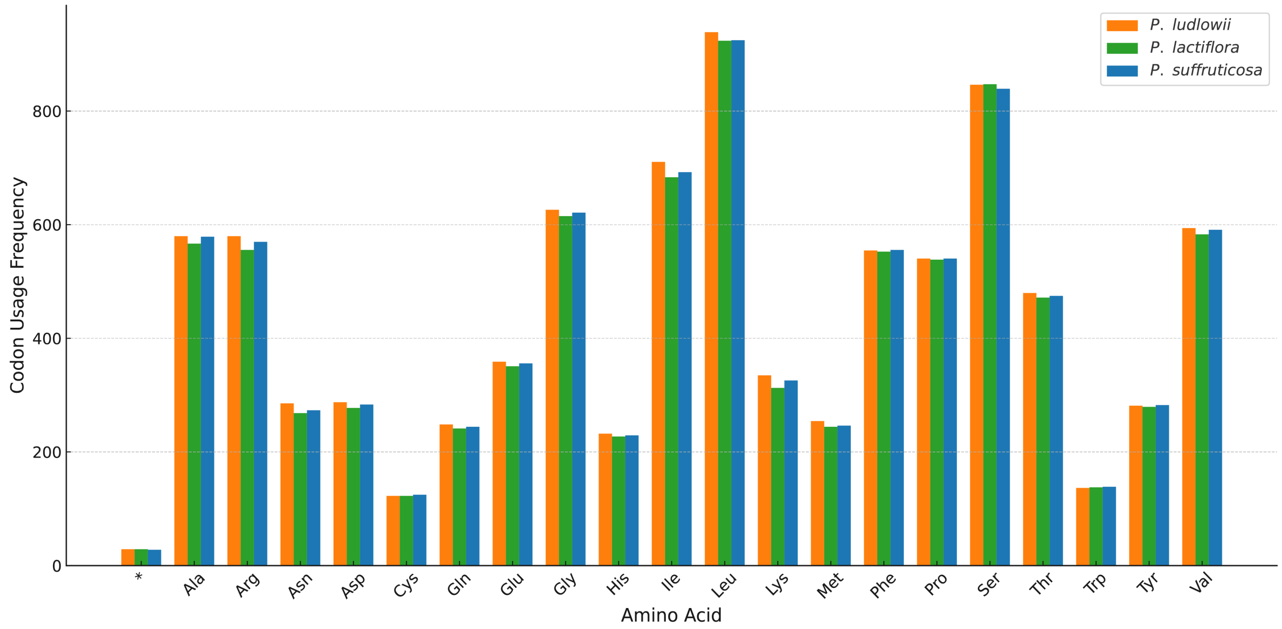

Figure S4 Codon usage frequency by amino acid across species. *: stands for stop codon

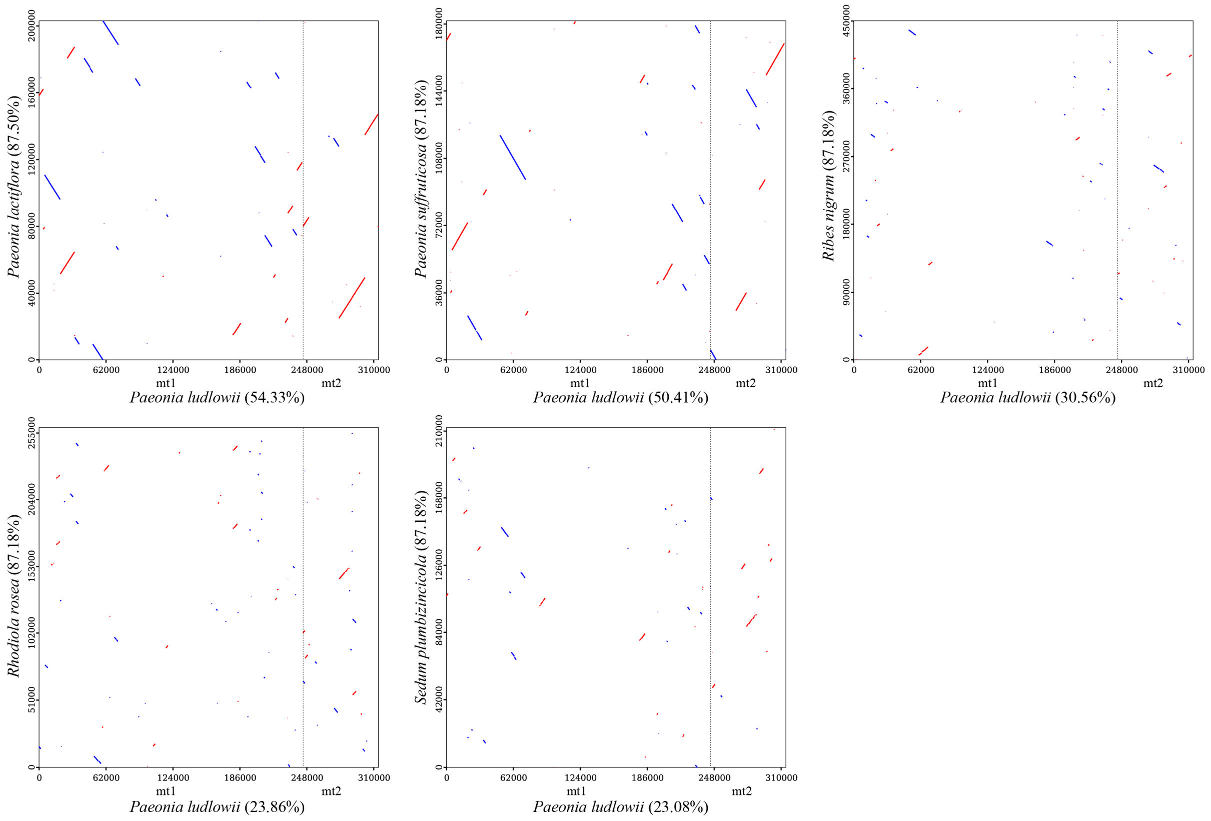

Figure S5 Dot plot of *P. ludlowii* with closely related species. The horizontal coordinate in each box indicates the assembled sequence, the vertical coordinate indicates the other sequences, the red line in the box indicates the forward comparison, and the blue line indicates the reverse complementary comparison.
